# A Mesophilic *Aeromonas salmonicida* Strain Isolated from an Unsuspected Host, the Migratory Bird Pied Avocet

**DOI:** 10.3390/microorganisms7120592

**Published:** 2019-11-20

**Authors:** Antony T. Vincent, Alex Bernatchez, Joachim Frey, Steve J. Charette

**Affiliations:** 1 INRS-Institut Armand-Frappier, Bacterial Symbionts Evolution, Laval City, QC H7V 1B7, Canada; antony.vincent@iaf.inrs.ca; 2 Institut de Biologie Intégrative et des Systèmes, Pavillon Charles-Eugène-Marchand, Université Laval, Quebec City, QC G1V 0A6, Canada; abernatchez1@outlook.com; 3 Centre de Recherche de l’Institut Universitaire de Cardiologie et de Pneumologie de Québec, Quebec City, QC G1V 4G5, Canada; 4 Département de Biochimie, de Microbiologie et de Bio-Informatique, Faculté des Sciences et de Génie, Université Laval; Quebec City, QC G1V 0A6, Canada; 5 Vetsuisse Faculty, University of Bern, CH-3001 Bern, Switzerland; joachim.frey@vetsuisse.unibe.ch

**Keywords:** *Aeromonas salmonicida*, mesophile, bird, pied avocet, *Dictyostelium discoideum*, pathogenesis

## Abstract

*Aeromonas salmonicida* is a Gram-negative bacterium, known as a fish pathogen since its discovery. Although the species was initially considered psychrophilic, a mesophilic subspecies (*pectinolytica*) and many other mesophilic strains still not attributed to subspecies have been described in the last two decades. These mesophilic strains were sampled from various sources, including humans, and some of them are known to be pathogenic. In this study, we describe a strain, JF2480, which was isolated from the spleen, and also found the kidney and liver of a dead pied avocet (*Recurvirostra avosetta*), a type of migratory bird inhabiting aquatic environments. A core genome phylogenomic analysis suggests that JF2480 is taxonomically distant from other known *A.* *salmonicida* subspecies. The genome sequence confirms that the strain possesses key virulence genes that are present in the typical *A. salmonicida* psychrophilic subspecies, with the exception of the genes encoding the type three secretion system (T3SS). Bacterial virulence assays conducted on the surrogate host *Dictyostelium discoideum* amoeba confirmed that the strain is virulent despite the lack of T3SS. Bacterial growth curves showed that strain JF2480 grow well at 40 °C, the body temperature of the pied avocet, and even faster at 41 °C, compared to other mesophilic strains. Discovery of this strain further demonstrates the extent of the phylogenomic tree of this species. This study also suggests that *A. salmonicida* can infect a wider array of hosts than previously suspected and that we need to rethink the way we perceive *A. salmonicida*’s natural environment.

## 1. Introduction

*Aeromonas salmonicida* is a Gram-negative bacterium, which is ubiquitous in aquatic environments. This species is divided into five officially recognized subspecies: *salmonicida*, *smithia*, *achromogenes*, *masoucida,* and *pectinolytica* [1]. Strains of the subspecies *salmonicida*, *smithia*, *achromogenes* and *masoucida* can infect a wide range of fish and are psychrophilic with their growth being limited to temperatures not higher than about 25 °C [1,2,3]. In 2000, the *pectinolytica* subspecies was isolated directly from a polluted river (Matanza River, Argentina) without having any known host [4]. This subspecies grows at 37 °C and is thus considered mesophilic. Some studies had previously reported the existence of mesophilic *A. salmonicida* strains, known as the hybridization group 3 [HG3], isolated from human and animal hosts [5,6,7,8]. However, the classification of these strains was made prior to the advent of DNA sequencing and we now know that biochemical approaches, compared to molecular ones, can sometimes lead to misclassifications for *A. salmonicida* [9]. Consequently, the finding of the subspecies *pectinolytica* was a major turning point for the taxonomy of *A. salmonicida* and suggested a greater diversity among the species of this bacterium.

Many new mesophilic *A. salmonicida* strains have been discovered and described especially in recent years. This was the case with the characterization of four Indian mesophilic *A. salmonicida* strains (Y47, Y567, Y577, A527) from undetermined subspecies and isolated from contaminated food [10,11,12]. Mesophilic *A. salmonicida* strains with human clinical background were also reported in Europe and India, including from a 68-year-old diabetic woman on continuous ambulatory peritoneal dialysis with abdominal pain and cloudy peritoneal fluid, in the blood of a 34-year-old female patient, from the skin infection of a 67-year-old immunocompetent male, in the right eye of a 55-year-old female who had recovered from cataract surgery, from a 15-year-old boy who had recovered from a finger surgery, from the feces of a child suffering from acute gastroenteritis, and finally from a person having cellulitis in a foot following trauma [13,14,15,16,17,18]. However, the pathogenicity of the isolates from human cases was not clearly demonstrated until a very recent study clearly showed the capacity of mesophilic *A. salmonicida* strains to infect mammals by doing infection experiments on mice [18].

The most recent phylogenomic analyses based on core genome revealed a great diversity in the mesophilic strains of the *A. salmonicida* species, opening the door to potentially more mesophilic subspecies than psychrophilic ones [11,18]. The increasing number of mesophilic strains also suggests the possibility to find other mesophilic *A. salmonicida* subspecies in unsuspected hosts.

In this study, we investigated the first case of bird infection by a mesophilic *A. salmonicida* strain. The strain was isolated from the spleen, and also found in the kidney and liver of a pied avocet (*Recurvirostra avosetta*) dead of sepsis. Pied avocets are migratory birds that feed in shallow lakes and mud ponds. The complete genome of this strain was sequenced and its growth capacity, virulence, and metabolic properties were investigated. This strain confirms the evolution of *A. salmonicida* species to infect three different classes of hosts (fish, mammals, and birds).

## 2. Materials and Methods

### 2.1. Bacterial Strains and Growth Conditions

*A. salmonicida* strains used in this study are described in Table 1. The strain that was retained and kept as JF2480 was an isolated colony from the spleen culture of a dead pied avocet. The spleen, liver, and kidney isolates were verified as *A. salmonicida* by phenotypic tests and sequencing *rrs* (16S rRNA), *gyrA*, and *rpoB* genes, but the latter two were not kept as they were identical to JF2480. When required for the experiments, the bacteria were thawed from glycerol 15% stock and grown on tryptic soy agar (TSA) for one day at 37 °C for the mesophilic strains, or three days at 18 °C for the *masoucida* subspecies.

### 2.2. DNA Extraction and Sequencing

The total genomic DNA of strain JF2480 was extracted using DNeasy Blood and Tissue kits (Qiagen, Montreal, QC, Canada) with the addition of a RNase treatment step to the manufacturer’s protocol. Sequencing was performed using a MiSeq (Illumina, San Diego, CA, USA) system at the Plateforme d’Analyse Génomique of the Institut de Biologie Intégrative et des Systèmes (Université Laval, Quebec City, QC, Canada). 

### 2.3. Sequence Assembly and Analyses

The sequencing reads were de novo assembled using A5-miseq version 20160825 [20]. The draft genome was annotated by Prokaryotic Genome Annotation Pipeline (PGAP) [21] and deposited in DDBJ/ENA/GenBank under the accession number VOIP00000000. The sequencing reads were deposited in SRA under the accession number PRJNA264317.

Molecular phylogeny was used to determine the phylogenetic position of strain JF2480 among the other *A. salmonicida*. To do this, the genomic sequence of strain JF2480 was added to a dataset comprising a representative of each of the *Aeromonas* species, all sequences from mesophilic *A. salmonicida* and the sequences of representatives of all psychrophilic subspecies of *A. salmonicida* (Table S1) [18]. All the 55 genome sequences were annotated using Prokka version 1.13.3 [22]. Homologous links between the translated coding sequences were found using the combination of the two algorithms COG [23] and OMCL [24] through GET_HOMOLOGUES version 20190102 [25]. The 2018 gene sequences (excluding paralogs) corresponding to the softcore (sequences present in more than 95% of the genomes) were aligned by codons using mafft version 7.407 [26] through TranslatorX version 1.1 [27]. The resulting alignments were filtered using BMGE version 1.12 [28] and concatenated in a partitioned supermatrix using AMAS [29]. The evaluation of the best-fit model of each partition (Table S2) and the maximum-likelihood phylogeny were done using IQ-TREE version 1.6.10 [30,31]. The robustness of the tree was assessed by performing 10,000 ultrafast bootstraps [32]. The final tree has been midpoint rooted using FigTree version 1.4.3 (http://tree.bio.ed.ac.uk/software/figtree/). The Average Nucleotide Identity (ANI) values were computed for genome sequences of *A. salmonicida* using pyani (https://github.com/widdowquinn/pyani). The percentage of conserved proteins (POCP) were computed for each pair of genomes using the OMCL algorithm [24] through GET_HOMOLOGUES version 20190102 [25]. Genes unique to JF2480 were found using PATRIC [33] and those involved in secretion systems with TXSScan [34]. EggNOG-mapper was used to further annotate the genes [35].

### 2.4. Virulence Assay

The virulence of *A. salmonicida* can be determined by its capacity to resist to the predation of *Dictyostelium discoideum* amoebae [36]. The strain DH1−10 of *D. discoideum* was grown in a Petri dish at 21 °C in HL5 liquid medium containing 15 µg/mL of tetracycline [37,38]. The *D. discoideum* cells were harvested, centrifuged, resuspended in HL5 without antibiotic, at the following concentrations: 100,000 cells/5 μL, 10,000 cells/5 μL, 1000 cells/5 μL, 100 cells/5 μL, 10 cells/5 μL and 0 cells/5 μL. Single colonies from *A. salmonicida* strains and from *Klebsiella aerogenes* strain, used as non-pathogenic control, were resuspended in 300 µL of tryptic soy broth (TSB) to an optical density of 0.4 at 595 nm (OD_595_) and 50 µL of the suspension were deposited in the wells of a 24-well plate containing 2 mL of HL5 agar. The dried bacterial lawns were spotted with 5 µL drops of *D. discoideum* cell suspension. The plates were incubated for 7 days at 18 °C and were then examined for the presence of phagocytic plaques [36].

### 2.5. Growth Curve

Bacteria from overnight growth on TSA at 37 °C (for all bacteria except *masoucida* subspecies grown at 18 °C) were resuspended in TSB. The turbidity was adjusted to an OD_595_ of 0.2, and the cultures were incubated in 48-well plate at 40 °C or 41 °C with shaking at 200 rpm in a Tecan Infinite F200 PRO microplate reader (Tecan, Morrisville, NC, USA). The ODs were measured automatically every 15 min for 12 h. Statistical significance between the growth curves was assessed using the compareGrowthCurves function of the R package statmod [39]. The *p*-values were adjusted for multiple testing using Holm’s method as implemented in compareGrowthCurves

## 3. Results

**Figure 1:**
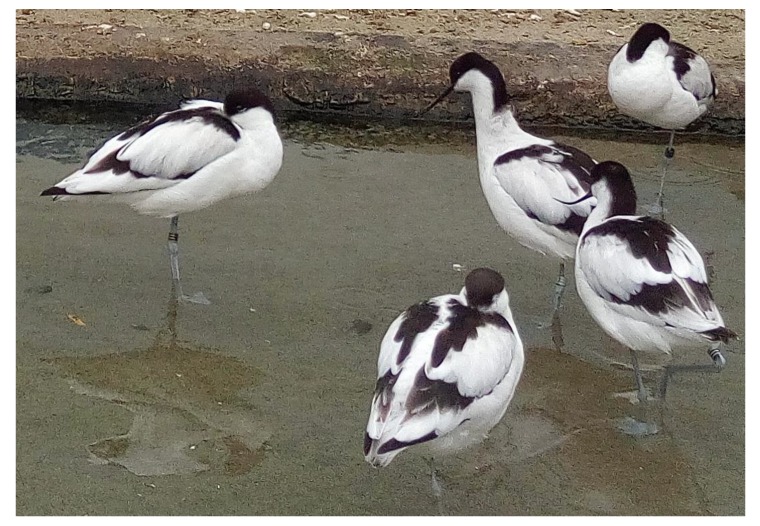
Pied avocets.

An *Aeromonas* sp. isolate initially identified as *Aeromonas hydrophila*-like strain was isolated in 1998 from a dead pied avocet (*Recurvirostra avosetta*) (Figure 1) that lived in a pied avocet colony in an outdoor enclosure of the zoo of Bern (Switzerland). The bird was the only dead one found in the colony of which the other individuals showed no signs of disease. It was removed rapidly after death and had undergone standard necropsy by a veterinary pathologist at the Department of Infectious Diseases and Pathobiology, approximately 3 h after death. Other than a grossly enlarged spleen, the tissues were reported free of macro-parasites and other lesions. Bacterial analysis was made immediately after the necropsy. The *Aeromonas* sp. was isolated abundantly as virtually pure culture from the spleen, kidney, and liver shortly after the death of the animal, indicating its role as causative agent of septicemia and death of the bird. An isolate from the spleen named JF2480, was conserved and further characterized based on *rrs* (16S rRNA) and *rpoB* sequence, which revealed that this bacterium was a member of the *A. salmonicida* species (data not shown). This was surprising since, to our knowledge, no case of bird infection by *A. salmonicida* has been reported so far. It was even more intriguing knowing that the body temperature of *Recurvirostra avosetta* is 40 °C [40], a relatively high temperature for this bacterial species.

**Figure 2:**
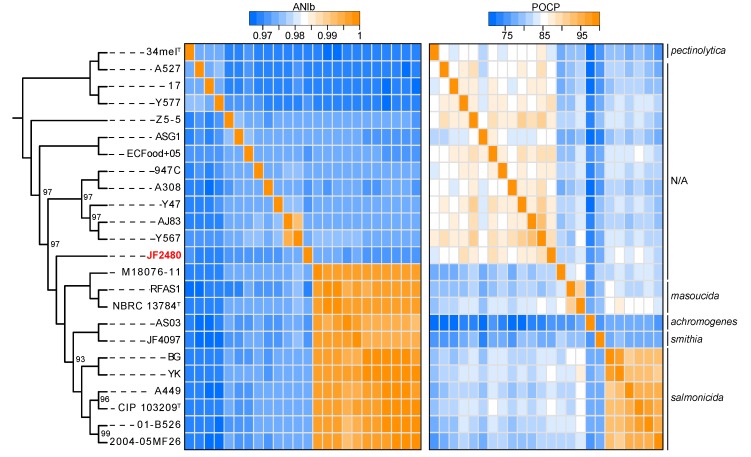
Cladogram showing the phylogenetic relations between JF2480 and other *A. salmonicida* strains. Only bootstrap values inferior to 100 are shown at the corresponding nodes. The heatmap represents the ANI values (in the middle) and the POCP values (on the right).

In order to have a clearer portrait of the identity of this bacterium, its genome was sequenced using the Illumina MiSeq technology. A robust phylogenomic analysis based on core genome was then performed to shed light on the phylogenetic relation between this unusual strain and the other *A. salmonicida* strains (Figure 2). Interestingly, its phylogenetic position is between the previously known mesophilic and psychrophilic strains. The ANI values confirm that it is, however, as distant as the previously known mesophilic strains, while all the psychrophilic strains are more closely related. To complement the ANI values, which permit us to measure the genetic distance in terms of nucleotide identity, the POCP values were also calculated. The POCP values, that reflect the level of conservation between different gene repertoires, clearly show that JF2480 is related to all other mesophilic strains. In addition to clustering all mesophilic strains together, the POCP values permit us to delineate the subspecies boundaries between psychrophilic subspecies [41]. Indeed, the different psychrophilic subspecies share ~99% nucleotide identity between them, making their separation difficult based on this criterion alone. However, POCPs have values around 80%, giving higher resolution than ANIs. This suggests a faster evolution of the gene repertoire in these subspecies compared to sequence evolution. This result is corroborated by various studies showing a high plasticity in the mobilome of *A. salmonicida* subsp. *salmonicida* and that this bacterium can easily acquire exogenous material by horizontal transfers [42,43,44].

Given the unusual host, the genome sequence was an opportunity to investigate the virulence factors of the strain JF2480. Secretion systems are protein machineries important for bacterial virulence [45]. The genome of strain JF2480 was predicted to possess mandatory genes for T1SS, T2SS, T4P, Tad, and T6SSi. Although no core genes encoding for T3SS have been detected, two genes coding for effectors (AexT and AexU), known to be secreted by this system, were found.

**Figure 3:**
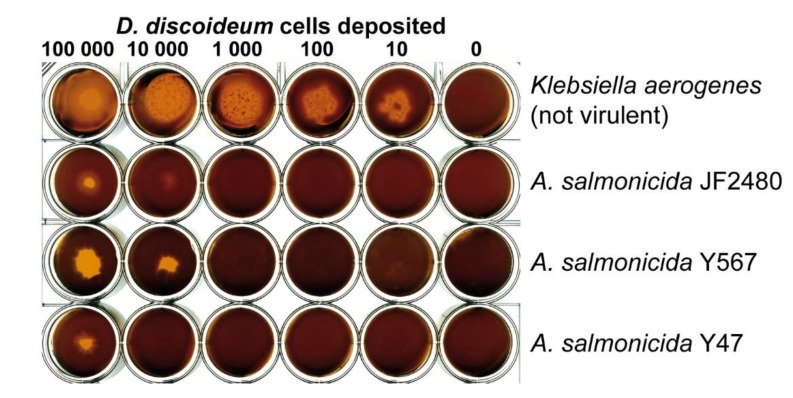
Virulence of strain JF2480 at low temperature. Different numbers of *D. discoideum* cells were deposited on a lawn of the different bacterial strains and grown on HL5 agar for 7 days at 18 °C. This test has been performed three times (biological replicates) giving similar results each time.

In the past, it has been shown that bacteria without a functional T3SS and especially *Aeromonas* bacteria were not virulent when tested in the predation assay involving the soil amoeba *D. discoideum* used as a surrogate host [36,46]. A low quantity of amoebae was able to create a phagocytic plaque on *K. aerogenes* (the positive control) while only a high quantity of *D. discoideum* cells was required to observe amoebal growth on JF2480 (Figure 3). Thus, bacterial virulence assays conducted on JF2480 revealed that this strain is virulent when facing *D. discoideum* even in absence of a complete T3SS compared to *K. aerogenes* bacterium known to be totally avirulent [47]. Other mesophilic *A. salmonicida* strains do not possess a complete T3SS. It is the case, among others, of the strains Y567 and Y47 isolated in a food market in India [10] (Table 1). These two strains have also displayed a clear virulence against *D. discoideum* (Figure 3).

A previous study that investigated the virulence of *A. salmonicida* from human clinical cases reported five genes uniquely found in mesophilic strains able to cause necrotizing fasciitis [18]. Two of these genes, encoding for a hemerythrin and a catalase, were confidently found in the genomes of JF2480, Y567, and Y47, which are virulent against *D. discoideum* (Table 2). Homologs of these two genes were already listed to be involved in the virulence of other bacteria [48,49].

Given the novelty of the host, it was relevant to investigate the genes unique to JF2480 to have clues about the genetic determinants that may be involved in its success of colonization. A total of 70 genes were found to be unique to JF2480 (Table S3). While several of them encode hypothetical proteins, others are related to mobile elements (phages and plasmids) and citrate metabolism. A gene encoding for a zonula occludens toxin-like (Zot) was also found. However, the gene is truncated in JF2480. By investigating the presence of this gene in other species of the genus *Aeromonas*, it has been possible to find that only *Aeromonas rivuli* has a homologue (Figure S1). The Zot toxin is known to be a virulence factor encoded in the genome of the lysogenic-filamentous phage CTXΦ that is integrated in the genome of the human pathogen *Vibrio cholerae* [50]. The phage CTXΦ also harbors genes producing the cholera toxin (*ctxA* and *ctxB*). These genes do not have clear homologs in the genome of JF2480, comparatively to the Zot toxin. Further research by performing TBLASTN analysis against a dataset containing representative genome sequences from all available *Aeromonas* species (Table S1), revealed a second gene encoding a putative Zot toxin, which this time seems complete. The distribution of this gene among *Aeromonas* is much more scattered than for the partial gene (Figure S1). For example, several mesophilic and psychrophilic strains of *A. salmonicida* possess an homologue of this gene.

**Figure 4:**
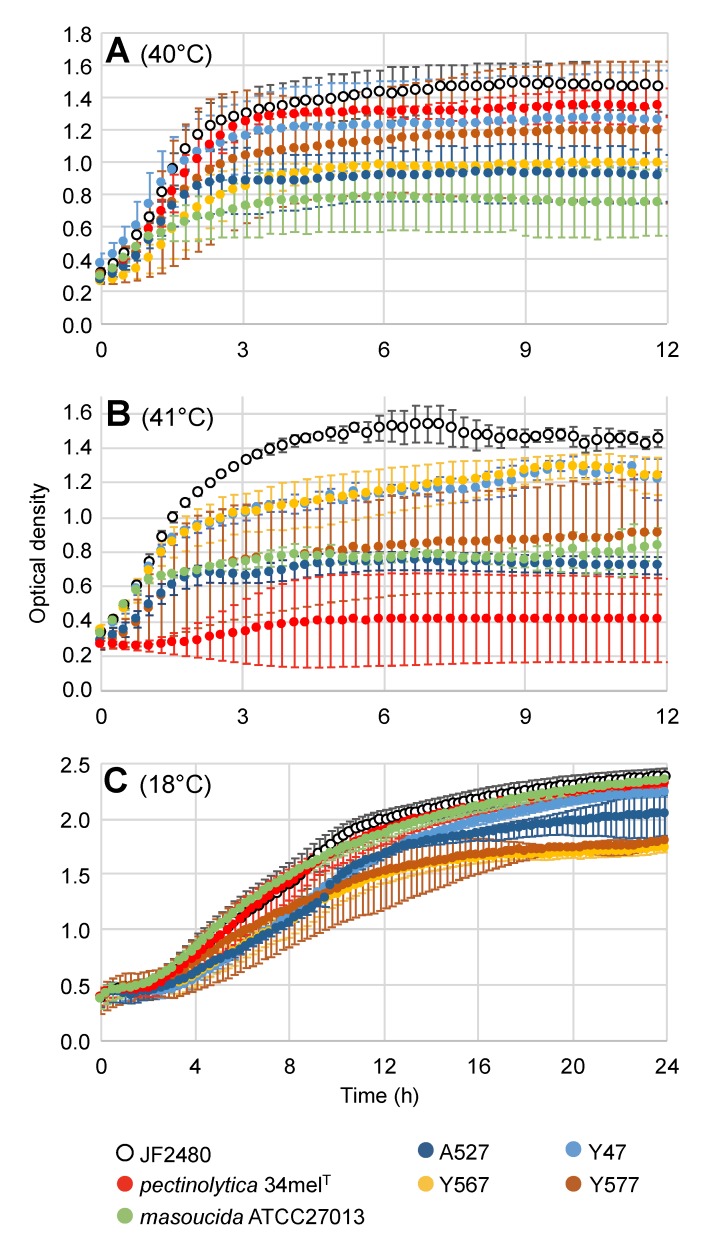
Growth curves for various *A. salmonicida* strains. The growth curves were determined at 40 °C (**A**), 41 °C (**B**) and 18 °C (**C**) by measuring the optical density at 595 nm. The means of two (**A**) and three biological replicates (**B** and **C**) are shown for each strain. Each experiment was also performed in three technical replicates.

The amoeba predation assay has been performed at 18 °C and not 37 °C because *D. discoideum* cannot survive over 26 °C. The virulence of strain JF2480 at this temperature suggests that the bacterium can grow quite well at low temperature. To confirm it, the growth curve of strain JF2480, as well as other *A. salmonicida* strains, was determined (Figure 4). It appears that JF2480 grew as well as the other strains tested at 18 °C. In addition, the strain JF2480 is the one with the best growth at 40 °C compared to other *A. salmonicida* mesophilic strains (Figure 4A), but this is not statistically significative (*p* = 1.0000 when comparing JF2480 with *masoucida* ATCC27013 for example). The difference in growth capacity is more pronounced at 41 °C, with strain JF2480 having the best growth compared to all other strains tested (Figure 4B). In this case, it is statistically significant (*p* < 0.0001 when comparing JF2480 with Y567 or Y47 which are the second-best growers). It is interesting to note that the strain from the *pectinolytica* subspecies showed good growth at 18 °C and 40 °C, but was much less important at 41 °C (*p* < 0.0001 when compared to A527). The other strains exhibited less fluctuating growth rate variations at the different temperatures. The growth of the *A. salmonicida* subspecies *masoucida* strain was one of the lowest at both 40 °C and 41 °C, which is coherent with previous results suggesting an intermediate lifestyle of this subspecies between psychrophilic and mesophilic [10].

Enzyme activities and details of the carbon sources utilized by the strain JF2480 are given in Tables S4 and S5. Extensive phenotypic characterization tests have been conducted demonstrating that strain JF2480 has a more diversified metabolism at 37 °C than 25 °C with additional carbon sources (D-manitol, D-glucose, palatinose, and D-sorbitol) that can be metabolized. However, even at 25 °C, the bacterium presented a wide range of metabolic activities.

## 4. Discussion

Historically, the bacterium *A. salmonicida* was known to infect salmonids, hence the origin of its name meaning “salmon killer” [51]. Since then, other bacteria belonging to *A. salmonicida* have been isolated from various fish species. In order to refine the taxonomy and respond to this diversity, different subspecies have been defined: *salmonicida*, *smithia*, *masoucida*, and *achromogenes*. The last subspecies to be defined, *pectinolytica*, made it possible to integrate into the *salmonicida* species an environmental and mesophilic bacterium, which can grow at a temperature of 37 °C, compared to other subspecies that are restricted to lower temperatures [4]. In recent years, several other mesophilic strains of *A. salmonicida* from the environment in India [10,11], and even human patients in Spain and Switzerland [14,18], have been isolated and characterized at the genomic level.

In this paper, a mesophilic strain of *A. salmonicida* isolated from a bird that died from septicemia, has been described and analyzed. The discovery of this strain shows that although the different strains are close evolutionarily (Figure 2), they have a wide range of hosts. The fact that psychrophilic strains are restricted to temperatures below 25 °C considerably limits the hosts that can be infected by them and are therefore specialized in different cold water fish. Mesophilic strains can grow at 37 °C (and even more, as shown with this study), and also at temperatures as low as those psychrophilic. This suggests that mesophilic strains have the potential to infect a wide range of hosts. This idea is supported by a previous study showing that mesophilic strains recovered from ill humans can also cause infection in mice [18]. Although strain JF2480 can be considered a pathogenic to *Recurvirostra avocetta* since it was abundantly present in 3 organs of a freshly dead bird, we do not know if the JF2480 strain is a polyvalent pathogen or if it is also, like the psychrophilic strains, specialized to specific hosts. On the other hand, the capacity of the JF2480 strain and other mesophilic strains to resist *D. discoideum* predation, a psychrophilic protozoan, opens the door to a wide range of hosts for mesophilic strains. Further infection experiments using mesophilic strains on fish will be mandatory in the future to help answer this question.

One of the reasons why the hosts that may be infected by mesophilic strains are still cryptic is that no strain has yet been isolated twice, thus making it impossible to determine confidently the real host range. Until very recently, *A. salmonicida* was mostly known as a pathogen of cold-water fish. It is therefore realistic to think that infections caused by these bacteria in other hosts have been falsely under-diagnosed. For example, we know that *A. salmonicida* can easily be diagnosed as *A. hydrophila* and that molecular analyses are needed to confirm the taxonomic identity of the strains with certainty [9,18].

In addition to demystifying their host range, the isolation of other mesophilic strains of *A. salmonicida* will shed light on their pathogenicity. As discussed elsewhere, T3SS is known to be a virulence factor essential to the pathogenicity of strains of the *salmonicida* subspecies [52]. Although T3SS seems to also be important for virulence of mesophilic strains [18], it is likely not essential as some virulent strains, as JF2480 described in the present study, lack it. Even without having a T3SS, JF2480 bears two effectors related to this system: AexT, a binary toxin with actin ADP-ribosylation activity and GAP activity toward Rho, Rac, and Cdc42 [53] and AexU. It is unclear if these effectors are the remnant of a previous T3SS or if they can be translocated by another secretion system. In *A. salmonicida* subsp. *salmonicida*, the T3SS locus is known to be located on a thermosensitive region that can be lost when strains are grown at a high temperature (>25 °C) [45]. It is still unclear if a former T3SS in JF2480 may have been lost in such a way.

As previously suggested [18], and reinforced by the present study, other genes also seem to be important for the virulence of mesophilic strains. These included five genes known to be virulence factors in other bacteria and potentially involved in necrotizing fasciitis by the mesophilic strains of *A. salmonicida* (Table 2). In the present study, we found two genes encoding zonula occludens toxin-like (Zot) in the genome of JF2480 (one partial and one likely complete), which is a virulence factor in *V. cholerae* [50]. In *V. cholerae*, Zot is encoded by the lysogenic phage CTXΦ along with the genes producing the cholera toxin. In the case of strain JF2480, the genes encoding Zot toxins are not predicted to be encoded on a mobile DNA element such as a prophage. In addition, genes encoding cholera toxin do not have homologues in JF2480. The fact that the partial gene is found at the moment only in strain JF2480 among *A. salmonicida,* suggests a recent acquisition horizontally, and not a vertical transfer. This is interesting in the context where it has been shown that the strains of the psychrophilic subspecies *A. salmonicida* subsp. *salmonicida* can acquire exogenous DNA from other pathogens, such as *Salmonella enterica* [42,43] and *Chlamydia suis* [44]. The present study suggests that some mesophilic strains of *A. salmonicida* may also adapt by acquiring genetic material from other pathogens and thus play a role in the dispersal of these genes. Regarding the likely complete gene, given its broad distribution among *Aeromonas* species (including other strains of *A. salmonicida*), it is likely not solely responsible for the bird infection, although it might have contributed as a virulence factor.

Several genes involved in citrate metabolism were also found. The use of citrate was already shown to be important for virulence of pathogens such as *Mycobacterium tuberculosis*, *Staphylococcus aureus,* and *V. cholerae* [54,55,56]. Although there is currently no evidence of such involvement in JF2480, this may be a clue to help decipher why these genes are present in this strain.

## 5. Conclusions

In conclusion, the discovery and description of the JF2480 strain, the first known *A. salmonicida* strain isolated from a bird, has become further support that this species can infect a wider array of hosts than previously suspected, and that we need to rethink the way we perceive *A. salmonicida*’s natural environment. In addition, the JF2480 strain is additional proof that the mesophilic group of the *salmonicida* species is more complex and diversified than the psychrophilic one. As already proposed [11,18], it is mandatory to continue this effort to isolate and describe new mesophilic *A. salmonicida* strains from different sources and hosts to finally have a global portrait of this bacterial species.

## Figures and Tables

**Table 1:** *A. salmonicida* strains used in this study.

Name	Subspecies	Lifestyle	Origin	Reference
JF2480	N/A	Mesophile	Switzerland	This study
Y47	N/A	Mesophile	India	[10]
Y567	N/A	Mesophile	India	[10]
Y577	N/A	Mesophile	India	[10]
A527	N/A	Mesophile	India	[11]
34 mel ^T^	*pectinolytica*	Mesophile	Argentina	[4]
NBRC 13784 ^T^	*masoucida*	Psychrophile	Japan	[19]

^T^: type strain.

**Table 2:** Presence of the five genes putatively involved in necrotizing fasciitis.

Protein	Virulence Trait	Strains^c^		
		JF2480	Y567	Y47
Two pore domain potassium channel family protein	N/A ^a^	40% ^d^	99%	99%
Hemerythrin	*A. hydrophila* survival in host macrophages	98%	98%	98%
Pseudaminic acid cytidylyltransferase	Colonisation of *H. pylori*	99%	50%^d^	100%
Catalase KatE ^b^	Virulence of *Leptospira* spp. in animal models	98%	99%	98%
UDP-N-acetylglucosamine-1-phosphate transferase ^c^	Production of enterobacterial antigen in *S. enterica*	45% ^d^	Absent	61% ^d^

a: N/A, none-applicable; b: The catalase was annotated as KatE by PATRIC [33]; c: % of similarity in regard to the virulent strain *A. salmonicida* 947 C; d: likely distant homologs.

## Supplementary Materials

The following are available online at https://www.mdpi.com/2076-2607/7/12/592/s1, Table S1. Genome sequences of *Aeromonas* used for the phylogenetic analysis; Table S2. Best-fit model of each partition; Table S3. Genes found to be unique to JF2480; Table S4. Phenotypic characterization of *A. salmonicida* strain JF2480 at 37 °C using a VITEK 2 system from bioMerieux; Table S5. Phenotypic characteristics of *A. salmonicida* strain JF2480 using a API bioMerieux apparatus; Figure S1. Distribution of the genes encoding the Zot toxin in the genus *Aeromonas* and percentage of similarity when present.
