# Supplementary material for: A Mesophilic Aeromonas salmonicida Strain Isolated from an Unsuspected Host, the Migratory Bird Pied Avocet
Source: Microorganisms. 2019 Nov 20;7(12):592. doi: 10.3390/microorganisms7120592 (PMC6955901; doi:10.3390/microorganisms7120592)
Supplement: Supplementary file 1 [file microorganisms-07-00592-s001.zip › Supplementary files/Supplementary information_Avocette_PROOFS-Final.docx]

**Supplementary information**

**A mesophilic *Aeromonas salmonicida* strain isolated from an unsuspected host, the migratory bird pied avocet**

Antony T. Vincent^1^, Alex Bernatchez^2,3,4^, Joachim Frey^5^ and Steve J. Charette^2,3,4^#

^1^ INRS-Institut Armand-Frappier, Bacterial Symbionts Evolution, Laval City, QC, Canada, H7V 1B7

^2^ Institut de biologie intégrative et des systèmes; Pavillon Charles-Eugène-Marchand; Université Laval; Quebec City, Quebec, G1V 0A6

^3^ Centre de recherche de l’Institut universitaire de cardiologie et de pneumologie de Québec; Hôpital Laval; Quebec City, Quebec, G1V 4G5

^4^ Département de biochimie, de microbiologie et de bio-informatique; Faculté des sciences et de génie; Université Laval; Quebec City, Quebec, G1V 0A6

^5^ Bern University, Bern, Switzerland, CH-3001

# Corresponding authors:

Steve J. Charette, Institut de Biologie Intégrative et des Systèmes, Charles-Eugène-Marchand, 1030 avenue de la Médecine, Université Laval, Quebec City, QC, Canada, G1V 0A6.
[Steve.Charette@bcm.ulaval.ca](mailto:Steve.Charette@bcm.ulaval.ca); Phone: 1-418-656-2131 ext. 406914 ; Fax: 418 948-5487

**Table S1. Genes found to be unique to JF2480.**

| **General Information** | | | | | | |  | **PATRIC** | | | |  | **EggNOG** | | | |
| --- | --- | --- | --- | --- | --- | --- | --- | --- | --- | --- | --- | --- | --- | --- | --- | --- |
| **ID** | **Start** | **End** | **Length** | **Strand** | **AA Length** | **Accession** |  | **PATRIC genus-specific families (PLfams)** | **PATRIC cross-genus families (PGfams)** | **Product** | **GO** |  | **bactNOG** | **Others** | **Funct-ional** | **Annotation** |
| 234 | 250533 | 251024 | 492 | - | 163 | 645.161.con.0001 |  | PLF_642_00007312 | PGF_00214872 | Histone acetyltransferase HPA2 and related acetyltransferases |  |  | bactNOG[38] | 05WQG@bactNOG,0QT7Z@gproNOG,17D4F@proNOG,COG0454@NOG | K | acetyltransferase |
| 624 | 8411 | 8632 | 222 | + | 73 | 645.161.con.0003 |  | PLF_642_00039450 | PGF_11292071 | hypothetical protein |  |  |  |  |  |  |
| 918 | 46302 | 46976 | 675 | - | 224 | 645.161.con.0004 |  | PLF_642_00029952 | PGF_07080421 | hypothetical protein |  |  | bactNOG[38] | 08MZJ@bactNOG,0QJ2M@gproNOG,101VF@NOG,1769F@proNOG | S | NA |
| 919 | 47157 | 47696 | 540 | + | 179 | 645.161.con.0004 |  | PLF_642_00030157 | PGF_07090978 | hypothetical protein |  |  | bactNOG[38] | 08W8T@bactNOG,0BNFM@bproNOG,11KIA@NOG,17B7P@proNOG | S | NA |
| 921 | 50622 | 51296 | 675 | + | 224 | 645.161.con.0004 |  |  | PGF_04852430 | hypothetical protein |  |  | bactNOG[38] | 05ZV4@bactNOG,09IBN@bctoNOG,128FQ@NOG | S | NA |
| 969 | 104022 | 104825 | 804 | + | 267 | 645.161.con.0004 |  | PLF_642_00032948 | PGF_08538531 | hypothetical protein |  |  | bactNOG[38] | 020KE@aproNOG,05T9T@bactNOG,11Q93@NOG,17JG7@proNOG | S | NA |
| 1510 | 5344 | 5853 | 510 | - | 169 | 645.161.con.0007 |  | PLF_642_00020357 | PGF_06549726 | Phage activator protein cII |  |  | bactNOG[38] | 05XR1@bactNOG,0QXBT@gproNOG,125BI@NOG,17MI4@proNOG | S | Phage regulatory protein CII |
| 1572 | 73736 | 74134 | 399 | - | 132 | 645.161.con.0007 |  | PLF_642_00020152 | PGF_04037129 | hypothetical protein |  |  | bactNOG[38] | 06YNJ@bactNOG,0R4TY@gproNOG,0YUH4@NOG,184QY@proNOG | S | NA |
| 1574 | 74783 | 75214 | 432 | + | 143 | 645.161.con.0007 |  | PLF_642_00009176 | PGF_00559254 | hypothetical protein |  |  |  |  |  |  |
| 1575 | 75243 | 75533 | 291 | - | 96 | 645.161.con.0007 |  | PLF_642_00011018 | PGF_00552274 | hypothetical protein |  |  |  |  |  |  |
| 1576 | 76085 | 76816 | 732 | - | 243 | 645.161.con.0007 |  | PLF_642_00013920 | PGF_00552031 | Zonula occludens toxin-like |  |  | bactNOG[38] | 08UST@bactNOG,0BCH5@bproNOG,11MTZ@NOG,17BM8@proNOG | S | Zonular occludens toxin |
| 1577 | 76850 | 77116 | 267 | - | 88 | 645.161.con.0007 |  | PLF_642_00032573 | PGF_08965383 | hypothetical protein |  |  |  |  |  |  |
| 1579 | 78350 | 78508 | 159 | - | 52 | 645.161.con.0007 |  | PLF_642_00034112 | PGF_09617610 | hypothetical protein |  |  |  |  |  |  |
| 1581 | 78641 | 78982 | 342 | - | 113 | 645.161.con.0007 |  | PLF_642_00016544 | PGF_00214949 | hypothetical protein |  |  |  |  |  |  |
| 1582 | 79173 | 80921 | 1749 | - | 582 | 645.161.con.0007 |  | PLF_642_00043231 | PGF_12931758 | hypothetical protein |  |  |  |  |  |  |
| 1584 | 81801 | 82625 | 825 | - | 274 | 645.161.con.0007 |  | PLF_642_00012163 | PGF_00551010 | hypothetical protein |  |  |  |  |  |  |
| 1875 | 197107 | 198726 | 1620 | + | 539 | 645.161.con.0008 |  | PLF_642_00007494 | PGF_01770932 | DNA phosphorothioation-dependent restriction protein DptF |  |  | bactNOG[38] | 08N2Z@bactNOG,0QNBX@gproNOG,1029M@NOG,174H3@proNOG | S | NA |
| 1876 | 198729 | 199613 | 885 | + | 294 | 645.161.con.0008 |  | PLF_642_00006901 | PGF_02239101 | DNA phosphorothioation-dependent restriction protein DptH / AAA-like domain |  |  | bactNOG[38] | 08B4Q@bactNOG,0QNMX@gproNOG,0XSZ8@NOG,174S8@proNOG | S | NA |
| 2001 | 148083 | 148253 | 171 | - | 56 | 645.161.con.0009 |  | PLF_642_00018095 | PGF_00554962 | hypothetical protein |  |  |  |  |  |  |
| 2034 | 191715 | 192740 | 1026 | + | 341 | 645.161.con.0009 |  | PLF_642_00011683 | PGF_00775359 | hypothetical protein |  |  |  |  |  |  |
| 2208 | 176289 | 176477 | 189 | - | 62 | 645.161.con.0010 |  | PLF_642_00023104 | PGF_05827274 | hypothetical protein |  |  |  |  |  |  |
| 2353 | 143354 | 145150 | 1797 | + | 598 | 645.161.con.0011 |  |  | PGF_00038305 | Uncharacterized DUF262-containing protein Tresu_1192 |  |  | bactNOG[38] | 07T1R@bactNOG,COG1479@NOG,COG3472@NOG | S | Pfam:DUF2081 |
| 2649 | 117864 | 118082 | 219 | - | 72 | 645.161.con.0013 |  | PLF_642_00010833 | PGF_00550062 | hypothetical protein |  |  |  |  |  |  |
| 2660 | 125790 | 125999 | 210 | - | 69 | 645.161.con.0013 |  | PLF_642_00006216 | PGF_00557884 | IncF plasmid conjugative transfer protein TraR |  |  | bactNOG[38] | 06BXS@bactNOG,0XW0K@NOG,17RWH@proNOG | S | Phage conjugal plasmid C-4 type zinc finger protein, TraR family |
| 2790 | 122901 | 123539 | 639 | + | 212 | 645.161.con.0014 |  | PLF_642_00012684 | PGF_06970560 | DNA-methyltransferase |  |  | bactNOG[38] | 05ECZ@bactNOG,0QJ4D@gproNOG,16S1X@proNOG,COG0338@NOG | L | D12 class N6 adenine-specific DNA methyltransferase |
| 2895 | 100667 | 102100 | 1434 | - | 477 | 645.161.con.0015 |  | PLF_642_00030354 | PGF_07268660 | hypothetical protein |  |  | bactNOG[38] | 08JQ6@bactNOG,173F1@proNOG,COG1123@NOG | S | ABC transporter |
| 2910 | 116778 | 117077 | 300 | - | 99 | 645.161.con.0015 |  | PLF_642_00009201 | PGF_00559273 | hypothetical protein |  |  |  |  |  |  |
| 3515 | 65968 | 66339 | 372 | + | 123 | 645.161.con.0022 |  | PLF_642_00027534 | PGF_04384631 | hypothetical protein |  |  |  |  |  |  |
| 3528 | 9377 | 11659 | 2283 | + | 760 | 645.161.con.0023 |  | PLF_642_00037364 | PGF_00025784 | Nitric-oxide reductase (EC 1.7.99.7), quinol-dependent |  |  | bactNOG[38] | 05DSQ@bactNOG,0B8CB@bproNOG,16RCX@proNOG,COG3256@NOG | P | Nitric oxide reductase |
| 3531 | 14985 | 15425 | 441 | - | 146 | 645.161.con.0023 |  | PLF_642_00031869 | PGF_08195007 | Phage protein |  |  |  |  |  |  |
| 3533 | 16155 | 16310 | 156 | + | 51 | 645.161.con.0023 |  | PLF_642_00018022 | PGF_00554890 | hypothetical protein |  |  |  |  |  |  |
| 3536 | 17306 | 17593 | 288 | - | 95 | 645.161.con.0023 |  | PLF_642_00007165 | PGF_00558302 | hypothetical protein |  |  |  |  |  |  |
| 3547 | 26376 | 26564 | 189 | + | 62 | 645.161.con.0023 |  | PLF_642_00030076 | PGF_00287171 | hypothetical protein |  |  | bactNOG[38] | 07Q23@bactNOG,0BQ7R@bproNOG,0ZUUX@NOG,16UD4@proNOG | S | NA |
| 3549 | 29687 | 30955 | 1269 | + | 422 | 645.161.con.0023 |  | PLF_642_00030565 | PGF_06247943 | Uncharacterized protein BT3327 |  |  | bactNOG[38] | 07QRD@bactNOG,0BGGF@bproNOG,17441@proNOG,COG2865@NOG | K | Transcriptional regulator |
| 3932 | 22357 | 27123 | 4767 | - | 1588 | 645.161.con.0030 |  | PLF_642_00007641 | PGF_00070054 | conserved hypothetical protein, putative DNA helicase |  |  | bactNOG[38] | 07RWC@bactNOG,0QISF@gproNOG,16RMN@proNOG,COG1112@NOG | L | DNA helicase |
| 3955 | 43478 | 43882 | 405 | + | 134 | 645.161.con.0030 |  | PLF_642_00007202 | PGF_00288534 | Mobile element protein |  |  | bactNOG[38] | 08PN6@bactNOG,0QPDN@gproNOG,0XS43@NOG,176QW@proNOG | L | Transposase |
| 3976 | 18303 | 18905 | 603 | + | 200 | 645.161.con.0031 |  | PLF_642_00023855 | PGF_10397226 | hypothetical protein |  |  | bactNOG[38] | 07965@bactNOG,0R5SV@gproNOG,0ZD5J@NOG,189FK@proNOG | S | NA |
| 3977 | 18905 | 19930 | 1026 | + | 341 | 645.161.con.0031 |  | PLF_642_00007952 | PGF_08089625 | hypothetical protein |  |  | bactNOG[38] | 06W2H@bactNOG,0R3JC@gproNOG,0YQQ3@NOG,1825D@proNOG | S | Pfam:GspK |
| 3978 | 19995 | 20870 | 876 | + | 291 | 645.161.con.0031 |  | PLF_642_00015916 | PGF_00111425 | hypothetical protein |  |  |  |  |  |  |
| 3979 | 20867 | 21463 | 597 | + | 198 | 645.161.con.0031 |  | PLF_642_00006779 | PGF_00375372 | hypothetical protein |  |  |  |  |  |  |
| 3980 | 21460 | 21957 | 498 | + | 165 | 645.161.con.0031 |  | PLF_642_00020345 | PGF_00148153 | hypothetical protein |  |  |  |  |  |  |
| 3983 | 26522 | 27100 | 579 | + | 192 | 645.161.con.0031 |  | PLF_642_00011964 | PGF_00550850 | hypothetical protein |  |  | bactNOG[38] | 05EVB@bactNOG,09DQM@bctoNOG,0FWU8@cytNOG,COG0110@NOG | S | acetyltransferase |
| 4074 | 26935 | 27768 | 834 | - | 277 | 645.161.con.0033 |  | PLF_642_00014834 | PGF_01189572 | Triphosphoribosyl-dephospho-CoA synthase (EC 2.4.2.52) |  |  | bactNOG[38] | 08K6F@bactNOG,0QMRJ@gproNOG,175CU@proNOG,COG1767@NOG | H | triphosphoribosyl-dephospho-CoA synthase |
| 4075 | 27746 | 28291 | 546 | - | 181 | 645.161.con.0033 |  |  | PGF_07518589 | Citrate lyase holo-[acyl-carrier-protein] synthase (EC 2.7.7.61) | GO:0050519\|holo-citrate lyase synthase activity |  | bactNOG[38] | 05YGP@bactNOG,0QRWM@gproNOG,179ZV@proNOG,COG3697@NOG | H, I | holo-ACP synthase CitX |
| 4076 | 28288 | 29808 | 1521 | - | 506 | 645.161.con.0033 |  | PLF_642_00014833 | PGF_00418097 | Citrate lyase alpha chain (EC 4.1.3.6) | GO:0008815\|citrate (pro-3S)-lyase activity |  | bactNOG[38] | 05CHY@bactNOG,0QHVJ@gproNOG,16R4J@proNOG,COG3051@NOG | C | citrate lyase, alpha |
| 4077 | 29819 | 30694 | 876 | - | 291 | 645.161.con.0033 |  | PLF_642_00014832 | PGF_01442763 | Citrate lyase beta chain (EC 4.1.3.6) | GO:0008815\|citrate (pro-3S)-lyase activity |  | bactNOG[38] | 05CI0@bactNOG,1AMYM@spiNOG,COG2301@NOG | C | Citrate lyase subunit beta |
| 4078 | 30691 | 30984 | 294 | - | 97 | 645.161.con.0033 |  | PLF_642_00014831 | PGF_00418101 | Citrate lyase gamma chain, acyl carrier protein |  |  | bactNOG[38] | 05XGS@bactNOG,0QUI3@gproNOG,17N2Q@proNOG,COG3052@NOG | C | Covalent carrier of the coenzyme of citrate lyase (By similarity) |
| 4079 | 31002 | 32021 | 1020 | - | 339 | 645.161.con.0033 |  | PLF_642_00014830 | PGF_00067742 | [Citrate [pro-3S]-lyase] ligase (EC 6.2.1.22) | GO:0008771\|[citrate (pro-3S)-lyase] ligase activity |  | bactNOG[38] | 05DUA@bactNOG,0QM0G@gproNOG,16TSA@proNOG,COG3053@NOG | C | (citrate (pro-3S)-lyase ligase |
| 4080 | 32040 | 32900 | 861 | - | 286 | 645.161.con.0033 |  | PLF_642_00014829 | PGF_04882920 | Fumarylacetoacetate hydrolase family protein |  |  | bactNOG[38] | 05ERH@bactNOG,0QKPU@gproNOG,16PQY@proNOG,COG0179@NOG | Q | fumarylacetoacetate (FAA) hydrolase |
| 4081 | 32928 | 34289 | 1362 | - | 453 | 645.161.con.0033 |  | PLF_642_00014828 | PGF_08034780 | Citrate-sodium symporter (TC 2.A.24.1.1) |  |  | bactNOG[38] | 05D2K@bactNOG,0QMK8@gproNOG,16T59@proNOG,COG3493@NOG | C | citrate carrier protein |
| 4082 | 34611 | 36272 | 1662 | + | 553 | 645.161.con.0033 |  | PLF_642_00020494 | PGF_03861808 | Tricarboxylate transport sensor protein TctE => Citrate response regulator CitA |  |  | bactNOG[38] | 05CEQ@bactNOG,0QI4X@gproNOG,16RTK@proNOG,COG3290@NOG | T | signal transduction histidine kinase regulating citrate malate metabolism |
| 4170 | 19390 | 19911 | 522 | - | 173 | 645.161.con.0036 |  | PLF_642_00010250 | PGF_02877841 | arabinogalactan endo-1,4-beta-galactosidase |  |  | bactNOG[38] | 06FKC@bactNOG,0QNW1@gproNOG,16QRC@proNOG,COG3867@NOG | G | arabinogalactan endo-1,4-beta-galactosidase |
| 4481 | 1294 | 1830 | 537 | - | 178 | 645.161.con.0049 |  | PLF_642_00020865 | PGF_06370515 | hypothetical protein |  |  | bactNOG[38] | 08YNI@bactNOG,11JCK@NOG | S | NA |
| 4482 | 2101 | 2448 | 348 | - | 115 | 645.161.con.0049 |  | PLF_642_00028968 | PGF_01524823 | hypothetical protein |  |  | bactNOG[38] | 06712@bactNOG,1244E@NOG | S | NA |
| 4485 | 8243 | 9184 | 942 | - | 313 | 645.161.con.0049 |  |  | PGF_00296314 | FIG00614279: hypothetical protein |  |  | bactNOG[38] | 06MV0@bactNOG,0R0V8@gproNOG,0YD4T@NOG,17Y4D@proNOG | S | NA |
| 4506 | 12047 | 12952 | 906 | + | 301 | 645.161.con.0050 |  |  | PGF_00055415 | TIR domain protein |  |  | bactNOG[38] | 090EB@bactNOG,11Q5J@NOG,175YM@proNOG | S | tir protein |
| 4509 | 1269 | 1829 | 561 | + | 186 | 645.161.con.0051 |  | PLF_642_00040652 | PGF_00169148 | hypothetical protein |  |  | bactNOG[38] | 06QWY@bactNOG,0R1W4@gproNOG,0YGUR@NOG,1801W@proNOG | S | NA |
| 4510 | 2604 | 3035 | 432 | + | 143 | 645.161.con.0051 |  | PLF_642_00029966 | PGF_07043081 | hypothetical protein |  |  | bactNOG[38] | 06BAC@bactNOG,0R64Y@gproNOG,0XYVF@NOG,1868R@proNOG | S | cell wall assembly cell proliferation coordinating protein, knr4-like protein |
| 4512 | 3521 | 3928 | 408 | + | 135 | 645.161.con.0051 |  | PLF_642_00038904 | PGF_12037504 | hypothetical protein |  |  |  |  |  |  |
| 4523 | 10213 | 10407 | 195 | + | 64 | 645.161.con.0051 |  | PLF_642_00025345 | PGF_02750863 | hypothetical protein |  |  |  |  |  |  |
| 4524 | 10481 | 10750 | 270 | + | 89 | 645.161.con.0051 |  | PLF_642_00043221 | PGF_11539613 | hypothetical protein |  |  |  |  |  |  |
| 4526 | 11046 | 11612 | 567 | + | 188 | 645.161.con.0051 |  | PLF_642_00025345 | PGF_02750863 | hypothetical protein |  |  |  |  |  |  |
| 4528 | 12603 | 12842 | 240 | + | 79 | 645.161.con.0051 |  | PLF_642_00037894 | PGF_12389040 | hypothetical protein |  |  |  |  |  |  |
| 4567 | 8516 | 9199 | 684 | + | 227 | 645.161.con.0054 |  |  | PGF_00366580 | hypothetical protein |  |  | bactNOG[38] | 07U04@bactNOG,0QNHI@gproNOG,0Y342@NOG,16V6I@proNOG | S | radical SAM domain protein |
| 4589 | 540 | 749 | 210 | + | 69 | 645.161.con.0058 |  | PLF_642_00006216 | PGF_00557884 | IncF plasmid conjugative transfer protein TraR |  |  | bactNOG[38] | 06BXS@bactNOG,0XW0K@NOG,17RWH@proNOG | S | Phage conjugal plasmid C-4 type zinc finger protein, TraR family |
| 4604 | 1288 | 1893 | 606 | + | 201 | 645.161.con.0061 |  | PLF_642_00025248 | PGF_06608536 | hypothetical protein |  |  | bactNOG[38] | 075DU@bactNOG,0R66T@gproNOG,0Z2UY@NOG,1878U@proNOG | S | NA |
| 4608 | 475 | 591 | 117 | - | 38 | 645.161.con.0063 |  | PLF_642_00005489 | PGF_00557619 | Putative endolysin or lysozyme (ACLAME 334) |  |  |  |  |  |  |
| 4612 | 1840 | 2049 | 210 | - | 69 | 645.161.con.0063 |  | PLF_642_00006216 | PGF_00557884 | IncF plasmid conjugative transfer protein TraR |  |  | bactNOG[38] | 06BXS@bactNOG,0XW0K@NOG,17RWH@proNOG | S | Phage conjugal plasmid C-4 type zinc finger protein, TraR family |
| 4617 | 1437 | 1556 | 120 | - | 39 | 645.161.con.0065 |  | PLF_642_00039551 | PGF_11729614 | hypothetical protein |  |  |  |  |  |  |
| 4649 | 397 | 996 | 600 | + | 200 | 645.161.con.0082 |  | PLF_642_00031807 | PGF_07838063 | Phage baseplate |  |  | bactNOG[38] | 05K1T@bactNOG,0QJSE@gproNOG,0ZQ4V@NOG,175K8@proNOG | S | phage Mu protein gp47-like protein |

**Table S2. Genome sequences of *Aeromonas* used for the phylogenetic analysis.**

| Species | Strain | GenBank | Reference |
| --- | --- | --- | --- |
| *A. allosaccharophila* | CECT 4199^T^ | NZ_CDBR00000000 | [1] |
| *A. aquatica* | AE235 | NZ_JRGL00000000 | [2] |
| *A. australiensis* | CECT 8023^T^ | NZ_CDDH00000000 | [1] |
| *A. bestiarum* | CECT 4227^T^ | NZ_CDDA00000000 | [1] |
| *A. bivalvium* | CECT 7113^T^ | NZ_CDBT00000000 | [1] |
| *A. cavernicola* | MDC 2508 | NZ_PGGC01000000 | [3] |
| *A. caviae* | 429865 Ae_01 | NZ_LIIX01000001 | [4] |
| *A. dhakensis* | AAK1 | NZ_BAFL00000000 | [5] |
| *A. diversa* | CDC 2478-85^T^ | NZ_APVG00000000 | [6] |
| *A. encheleia* | CECT 4342^T^ | NZ_CDDI00000000 | [1] |
| *A. enteropelogenes* | CECT 4255^T^ | NZ_CDDE00000000 | [1] |
| *A. eucrenophila* | CECT 4224^T^ | NZ_CDDF00000000 | [1] |
| *A. finlandiensis* | 4287D | NZ_JRGK00000000 | [7] |
| *A. fluvialis* | LMG 24681^T^ | NZ_CDBO00000000 | [1] |
| *A. hydrophila* | ATCC 7966^T^ | NC_008570 | [8] |
| *A. jandaei* | CECT 4228^T^ | NZ_CDBV00000000 | [1] |
| *A. lacus* | AE122 | NZ_JRGM00000000 | [7] |
| *A. lusitana* | MDC 2473 | PGCP01000000 | [9] |
| *A. media* | WS | NZ_CP007567, NZ_CP007568 | [10] |
| *A. molluscorum* | 848^T^ | NZ_AQGQ00000000 | [11] |
| *A. piscicola* | LMG 24783^T^ | NZ_CDBL00000000 | [1] |
| *A. popoffii* | CIP 105493^T^ | NZ_CDBI00000000 | [1] |
| *A. rivipollensis* | KN-Mc-11N1 | NZ_CP027856 | [12] |
| *A. rivuli* | DSM 22539^T^ | NZ_CDBJ01000000 | [1] |
| *A. sanarellii* | LMG 24682^T^ | NZ_CDBN00000000 | [1] |
| *A. schubertii* | WL1483 | NZ_CP013067 | [13] |
| *A. simiae* | CIP 107798 | NZ_CDBY00000000 | [1] |
| *A. sobria* | CECT 4245^T^ | NZ_CDBW01000000 | [1] |
| *A. taiwanensis* | LMG 24683^T^ | NZ_BAWK00000000 | [14] |
| *A. tecta* | CECT 7082^T^ | NZ_CDCA00000000 | [1] |
| *A. veronii* | B565 | NC_015424 | [15] |
| *A. salmonicida* subsp. *salmonicida* | 01-B526 | NZ_CP027000, KY555069, KY555070 | [16] |
| *A. salmonicida* subsp. *salmonicida* | 2004-05MF26 | JRYW00000000 | [17] |
| *A. salmonicida* subsp. *salmonicida* | A449 | CP000644, P000645, CP000646 | [18] |
| *A. salmonicida* subsp. *salmonicida* | CIP 103209^T^ | CDDW00000000 | [1] |
| *A. salmonicida* subsp. *salmonicida* | BG | LUHO00000000 | [19] |
| *A. salmonicida* subsp. *salmonicida* | YK | LUHP00000000 | [19] |
| *A. salmonicida* subsp. *achromogenes* | AS03 | AMQG00000000 | [20] |
| *A. salmonicida* subsp. *smithia* | JF4097 | JZTI00000000 | [21] |
| *A. salmonicida* subsp. *masoucida* | NBRC 13784^T^ | BAWQ01000000 | N/A^a^ |
| *A. salmonicida* subsp. *masoucida* | RFAS1 | NZ_CP017143 | [22] |
| *A. salmonicida* | M18076-11 | NQMJ00000000 | [23] |
| *A. salmonicida* | Y47 | JZTF00000000 | [21] |
| *A. salmonicida* | Y567 | JZTG00000000 | [21] |
| *A. salmonicida* | Y577 | JZTH00000000 | [21] |
| *A. salmonicida* | A527 | CP022550 | [24] |
| *A. salmonicida* | ECFood+05 | NZ_NVQH01000000 | N/A^a^ |
| *A. salmonicida* | ASG1 | PRJNA377399 | [25] |
| *A. salmonicida* | A308 | PSZJ00000000 | [26] |
| *A. salmonicida* | 947C | PSZK00000000 | [26] |
| *A. salmonicida* | AJ83 | PSZI00000000 | [26] |
| *A. salmonicida* | 17 | NZ_QLLM00000000 | N/A^a^ |
| *A. salmonicida* | Z5-5 | NZ_NXBT00000000 | [27] |
| *A. salmonicida* | JF2480 | VOIP00000000 | This study |
| *A. salmonicida* subsp. *pectinolytica* | 34mel^T^ | NZ_CP022426 | [28] |

a : not applicable (N/A)

**Table S3. Phenotypic characterization of *A. salmonicida* strain JF2480 at 37°C using a VITEK 2 system from bioMerieux.** Data analyzed according to manufacturer instructions. +, positive; -, negative.

| D-glucose fermentation | + |  | lipase | + |
| --- | --- | --- | --- | --- |
| urease | + |  | palatinose assimilation | + |
| β-galactosidase | + |  | tyrosine arylamidase | + |
| D-mannose assimilation | + |  | D-sorbitol assimilation | + |
| D-mannitol assimilation | + |  | saccharose sucrose assimilation | + |
| D-maltose assimilation | + |  | D-tagatose assimilation | - |
| β-glucuronidase | - |  | D-trehalose assimilation | + |
| L-malate assimilation | - |  | α-glucosidase | - |
| citrate assimilation | + |  | succinate alkalinization | + |
| Ala-Phe-Pro arylamidase | + |  | β-N-acetyl-galactosaminidase | - |
| adonitol assimilation | - |  | α-galactosidase | - |
| L-arabitol assimilation | - |  | ornitine-decarboxylase | - |
| L-pyrrolydonyl-arylamidase | - |  | lysine decarboxylase | - |
| D-cellobiose | + |  | coumarate assimilation | + |
| H_2_S production | - |  | malonate assimilation | - |
| β-N-acetyl glucosaminidase | + |  | 5-keto-D-gluconat assimilation | - |
| glutamyl-arylamidase | - |  | L-lactate alcalinization | - |
| γ-glytamyl-transferase | - |  | L-lactate assimilation | - |
| β-glucosidase | + |  | Phosphatase | - |
| β-xylosidase | - |  | Glycin arylamidase | - |
| β-alanine arylamidase | - |  | L-hystidin assimilation | - |
| L-proline arylamidase | + |  | Glu-Gly-Arg arylamidase | + |
| Glu-Gly-Arg arylamidase | + |  |  |  |

**Table S4. Phenotypic characteristics of *A. salmonicida* strain JF2480 using a API® bioMerieux apparatus.** Data analyzed according to manufacturer instructions and read after 48 h incubation at 25 °C and 37 °C. +, positive; -, negative.

| **Test** | **25°C** | **37°C** |
| --- | --- | --- |
| L-ornitine | **-** | **-** |
| L-Arginin | **+** | **-** |
| L-Lysin | **-** | **-** |
| Urea | **-** | **-** |
| L-Arabitol | **-** | **-** |
| Galacturonic acid | **-** | **-** |
| Potassium5-ketogluconate | **-** | **-** |
| 5-Bromo-3-indoxyl-nonanoat (Lipase) | **+** | **+** |
| Sodiumpyruvate | **-** | **-** |
| 4-Nitrophenyl-βD-glycopyranosid (β-Glucosidase) | **+** | **+** |
| D-manitol | **-** | **+** |
| D-maltose | **+** | **+** |
| L-Tryptophan (tryptophanase) | **+** | **+** |
| 5-bromo-4-chloro-3-indolyl-N-acetyl-βD-glucosamide | **+** | **+** |
| 4-Nitrophenyl-βD-galactopyranosid (β-Galactosidase) | **+** | **+** |
| D-Glucose | **-** | **+** |
| D-saccharose | **+** | **+** |
| L-Arabinose | **+** | **+** |
| D-Arabitol | **-** | **-** |
| 4-Nitrophenyl-αD-glycopyranosid (α-Glucosidase) | **-** | **-** |
| 4-Nitrophenyl-αD-galactopyranosid (α-Galactosidase) | **-** | **-** |
| D-Trehalose | **+** | **+** |
| l-Rhamnose | **-** | **-** |
| Inositol | **-** | **-** |
| Adonitol | **-** | **-** |
| Palatinose | **-** | **+** |
| 4-Nitrophenyl-βD-glucuronid (β-Glucuronidase) | **-** | **-** |
| D-Cellobiose | **+** | **+** |
| D-Sorbitol | **-** | **+** |
| 4-Nitrophenyl-αD-maltopyranosid (α-Maltosidase) | **-** | **-** |
| Sodiummalonat | **-** | **-** |
| L-Aspartat-4-notroanilid (L-Aspartat-Arylamidase) | **-** | **-** |

**Table S5.** Best-fit model of each partition

For this table, please see the Excel file also found in the Supplementary information of this manuscript.

**Figure S1.** **Distribution of the genes encoding the Zot toxin in the genus *Aeromonas* and percentage of similarity when present.**

References

1. Colston, S.M.; Fullmer, M.S.; Beka, L.; Lamy, B.; Gogarten, J.P.; Graf, J. Bioinformatic genome comparisons for taxonomic and phylogenetic assignments using aeromonas as a test case. *mBio* **2014**, *5*, e02136.

2. Hossain, M.J.; Beaz-Hidalgo, R.; Figueras, M.J.; Liles, M.R. Draft genome sequences of two novel aeromonas species recovered in association with cyanobacterial blooms. *Genome announcements* **2014**, *2*.

3. Martinez-Murcia, A.; Beaz-Hidalgo, R.; Svec, P.; Saavedra, M.J.; Figueras, M.J.; Sedlacek, I. Aeromonas cavernicola sp. Nov., isolated from fresh water of a brook in a cavern. *Current microbiology* **2013**, *66*, 197-204.

4. Padilla, J.C.; Bustos, P.; Castro-Escarpulli, G.; Sanchez-Varela, A.; Palma-Martinez, I.; Arzate-Barbosa, P.; Garcia-Perez, C.A.; Lopez-Lopez Mde, J.; Gonzalez, V.; Guo, X. Draft genome sequence of aeromonas caviae strain 429865 inp, isolated from a mexican patient. *Genome announcements* **2015**, *3*.

5. Wu, C.J.; Wang, H.C.; Chen, C.S.; Shu, H.Y.; Kao, A.W.; Chen, P.L.; Ko, W.C. Genome sequence of a novel human pathogen, aeromonas aquariorum. *Journal of bacteriology* **2012**, *194*, 4114-4115.

6. Farfan, M.; Spataro, N.; Sanglas, A.; Albarral, V.; Loren, J.G.; Bosch, E.; Fuste, M.C. Draft genome sequence of the aeromonas diversa type strain. *Genome announcements* **2013**, *1*.

7. Beaz-Hidalgo, R.; Latif-Eugenin, F.; Hossain, M.J.; Berg, K.; Niemi, R.M.; Rapala, J.; Lyra, C.; Liles, M.R.; Figueras, M.J. Aeromonas aquatica sp. Nov., aeromonas finlandiensis sp. Nov. And aeromonas lacus sp. Nov. Isolated from finnish waters associated with cyanobacterial blooms. *Systematic and applied microbiology* **2015**, *38*, 161-168.

8. Seshadri, R.; Joseph, S.W.; Chopra, A.K.; Sha, J.; Shaw, J.; Graf, J.; Haft, D.; Wu, M.; Ren, Q.; Rosovitz, M.J.*, et al.* Genome sequence of aeromonas hydrophila atcc 7966t: Jack of all trades. *Journal of bacteriology* **2006**, *188*, 8272-8282.

9. Martinez-Murcia, A.; Beaz-Hidalgo, R.; Navarro, A.; Carvalho, M.J.; Aravena-Roman, M.; Correia, A.; Figueras, M.J.; Saavedra, M.J. Aeromonas lusitana sp. Nov., isolated from untreated water and vegetables. *Current microbiology* **2016**, *72*, 795-803.

10. Chai, B.; Wang, H.; Chen, X. Draft genome sequence of high-melanin-yielding aeromonas media strain ws. *Journal of bacteriology* **2012**, *194*, 6693-6694.

11. Spataro, N.; Farfan, M.; Albarral, V.; Sanglas, A.; Loren, J.G.; Fuste, M.C.; Bosch, E. Draft genome sequence of aeromonas molluscorum strain 848tt, isolated from bivalve molluscs. *Genome announcements* **2013**, *1*.

12. Park, S.Y.; Lim, S.R.; Son, J.S.; Kim, H.K.; Yoon, S.W.; Jeong, D.G.; Lee, M.S.; Lee, J.R.; Lee, D.H.; Kim, J.H. Complete genome sequence of aeromonas rivipollensis kn-mc-11n1, isolated from a wild nutria (myocastor coypus) in south korea. *Microbiology resource announcements* **2018**, *7*.

13. Liu, L.; Li, N.; Zhang, D.; Fu, X.; Shi, C.; Lin, Q.; Hao, G. Complete genome sequence of the highly virulent aeromonas schubertii strain wl1483, isolated from diseased snakehead fish (channa argus) in china. *Genome announcements* **2016**, *4*.

14. Wang, H.C.; Ko, W.C.; Shu, H.Y.; Chen, P.L.; Wang, Y.C.; Wu, C.J. Genome sequence of aeromonas taiwanensis lmg 24683t, a clinical wound isolate from taiwan. *Genome announcements* **2014**, *2*.

15. Li, Y.; Liu, Y.; Zhou, Z.; Huang, H.; Ren, Y.; Zhang, Y.; Li, G.; Zhou, Z.; Wang, L. Complete genome sequence of aeromonas veronii strain b565. *Journal of bacteriology* **2011**, *193*, 3389-3390.

16. Vincent, A.T.; Charette, S.J. Completion of genome of aeromonas salmonicida subsp. Salmonicida 01-b526 reveals how sequencing technologies can influence sequence quality and result interpretations. *New microbes and new infections* **2018**, *25*, 24-26.

17. Vincent, A.T.; Tanaka, K.H.; Trudel, M.V.; Frenette, M.; Derome, N.; Charette, S.J. Draft genome sequences of two aeromonas salmonicida subsp. Salmonicida isolates harboring plasmids conferring antibiotic resistance. *FEMS microbiology letters* **2015**, *362*.

18. Reith, M.E.; Singh, R.K.; Curtis, B.; Boyd, J.M.; Bouevitch, A.; Kimball, J.; Munholland, J.; Murphy, C.; Sarty, D.; Williams, J.*, et al.* The genome of aeromonas salmonicida subsp. Salmonicida a449: Insights into the evolution of a fish pathogen. *BMC genomics* **2008**, *9*, 427.

19. Long, M.; Nielsen, T.K.; Leisner, J.J.; Hansen, L.H.; Shen, Z.X.; Zhang, Q.Q.; Li, A. Aeromonas salmonicida subsp. Salmonicida strains isolated from chinese freshwater fish contain a novel genomic island and possible regional-specific mobile genetic elements profiles. *FEMS microbiology letters* **2016**, *363*.

20. Han, J.E.; Kim, J.H.; Shin, S.P.; Jun, J.W.; Chai, J.Y.; Park, S.C. Draft genome sequence of *aeromonas salmonicida* subsp. *Achromogenes* as03, an atypical strain isolated from crucian carp (*carassius carassius*) in the republic of korea. *Genome announcements* **2013**, *1*, e00791-00713.

21. Vincent, A.T.; Trudel, M.V.; Freschi, L.; Nagar, V.; Gagne-Thivierge, C.; Levesque, R.C.; Charette, S.J. Increasing genomic diversity and evidence of constrained lifestyle evolution due to insertion sequences in *aeromonas salmonicida*. *BMC genomics* **2016**, *17*, 44.

22. Han, H.J.; Kim, D.Y.; Kim, W.S.; Kim, C.S.; Jung, S.J.; Oh, M.J.; Kim, D.H. Atypical aeromonas salmonicida infection in the black rockfish, sebastes schlegeli hilgendorf, in korea. *Journal of fish diseases* **2011**, *34*, 47-55.

23. Rouleau, F.D.; Vincent, A.T.; Charette, S.J. Genomic and phenotypic characterization of an atypical aeromonas salmonicida strain isolated from a lumpfish and producing unusual granular structures. *Journal of fish diseases* **2018**, *41*, 673-681.

24. Vincent, A.T.; Rouleau, F.D.; Moineau, S.; Charette, S.J. Study of mesophilic *aeromonas salmonicida* a527 strain sheds light on the species' lifestyles and taxonomic dilemma. *FEMS microbiology letters* **2017**, *364*, fnx239.

25. Ruppe, E.; Cherkaoui, A.; Wagner, N.; La Scala, G.C.; Beaulieu, J.Y.; Girard, M.; Frey, J.; Lazarevic, V.; Schrenzel, J. In vivo selection of a multidrug-resistant aeromonas salmonicida during medicinal leech therapy. *New microbes and new infections* **2018**, *21*, 23-27.

26. Vincent, A.T.; Fernandez-Bravo, A.; Sanchis, M.; Mayayo, E.; Figueras, M.J.; Charette, S.J. Investigation of the virulence and genomics of *aeromonas salmonicida* strains isolated from human patients. *Infection, genetics and evolution : journal of molecular epidemiology and evolutionary genetics in infectious diseases* **2019**, *68*, 1-9.

27. Shen, Y.; Xu, C.; Sun, Q.; Schwarz, S.; Ou, Y.; Yang, L.; Huang, Z.; Eichhorn, I.; Walsh, T.R.; Wang, Y.*, et al.* Prevalence and genetic analysis of mcr-3-positive aeromonas species from humans, retail meat, and environmental water samples. *Antimicrobial agents and chemotherapy* **2018**, *62*.

28. Pavan, M.E.; Pavan, E.E.; Lopez, N.I.; Levin, L.; Pettinari, M.J. Living in an extremely polluted environment: Clues from the genome of melanin-producing aeromonas salmonicida subsp. Pectinolytica 34melt. *Applied and environmental microbiology* **2015**, *81*, 5235-5248.
